# Increasing Physical Exercise through Action and Coping Planning

**DOI:** 10.3390/ijerph19073883

**Published:** 2022-03-24

**Authors:** Zhi Qiang Clement Wee, Denise Dillon

**Affiliations:** School of Social and Health Sciences, James Cook University Singapore, Singapore 387380, Singapore; clement.wee@clarity-singapore.org

**Keywords:** intention-behaviour gap, action planning, coping planning, increase in physical exercise, habits

## Abstract

The intention-behaviour gap has been a barrier to health behavioural change. A total of 85 participants (aged 18–56 years) were recruited for a study that examined how two types of planning (Action and Coping) could bridge the intention-behaviour gap and increase physical exercise behaviours. The online study took place over two weeks, with participants completing pre- and post- measures of past exercise habits, intentions to exercise, subsequent physical exercise behaviours, intrinsic motivation levels, and engagement in action and coping planning. A hierarchical regression analysis showed that intentions, past exercise habits and action planning were significant predictors of change in physical exercise behaviours. Positive correlations were observed between participants’ past habits and their exercise behaviours during the study. 71.8% of participants met the World Health Organization’s (WHO) recommended guidelines for physical activity at the end of the study compared to the initial 58.8%, which evidences a significant increase in participant physical exercise behaviours. Our findings revealed that while intentions are important predictors for behavioural change, cultivating habits to engage in regular exercise seems to outweigh the significance of intentions. Moreover, action planning could be a helpful intervention to bridge the intention-behaviour gap to increase overall physical exercise behaviours. In the long-term, this would improve an individual’s mental and physical wellbeing and potentially alleviate the costly burden on public health services.

## 1. Introduction

Physical inactivity results in a costly and problematic burden on public health. The World Health Organisation (WHO) recommends that individuals between the ages of 18–65 should engage in at least 150 min of moderate to intense physical activity during the week, or at least 75 min of vigorous-intensity physical activity, or an equivalent combination of both. A WHO 2018 study showed that 1.4 billion adults are physically inactive and are at risk of developing or exacerbating problems such as obesity, cardiovascular disease, diabetes, hypertension and cancer [1]. As such, many studies have explored and confirmed the physiological and psychological long-term benefits of physical activity in hopes to increase buy-in [2] or to create cost-effective strategies to increase physical activity in individuals [3]. Physiological and psychological benefits in combination also reduce the risk of developing common mental health illnesses such as depression and anxiety [1,4]. 

Given the benefits of physical activity and the status of large portions of the population being insufficiently active, it remains important to consider how physical activity may be promoted in individuals such that more individuals meet the recommended physical activity guidelines. This could be through increasing the duration, intensity, or frequency at which one engages in physical exercise, or even simply to begin engaging in regular physical exercise behaviours. 

The intention-behaviour gap refers to the discrepancy between individuals developing the intention to engage in a behaviour and actually taking up the action [5,6]. Physical inactivity is particularly prevalent in high-income Asia Pacific regions such as Singapore with approximately one in three participants (i.e., 36.5%) with insufficient physical activity as compared to global figures of one in four (i.e., 27.5%) [1]. The prevalence was observed to have increased over the past two decades, while prevalence rates remained stable across other countries [1]. Despite a large proportion of Singaporeans agreeing or strongly agreeing that wellbeing benefits of engaging in physical exercise can be physical, mental, and emotional health, many fail to regularly engage in physical exercise [7], with major barriers to engaging in physical activity being lack of time or interest. Less frequently reported barriers such as lack of a sporting partner, nearby facilities or money for sports activities indicate at least some intention could be present. It appears that one pathway to increasing physical exercise levels in Singaporeans is to bridge the intention-behaviour gap.

Planning is a strategy that has been found to enable an increase in individuals’ physical activity levels. Planning is defined as a self-regulatory strategy which adopts the use of mental stimulation to help individuals mentally link their behavioural responses to future situations [8]. This self-regulatory strategy is proposed to in both the Health Action Process Approach (HAPA) [9] and the transtheoretical model (TTM) of health behaviour change [10]. The HAPA depicts two different stages in the health-behaviour change process (motivational & volitional stages); the TTM has six stages. The HAPA and TTM are aligned in the following areas: (1) The setting and development of intentions lie with the motivational phase (HAPA) and the contemplation stage (TTM). (2) The volitional stage (HAPA) describing the steps that people take to translate intentions into actions through planning can be seen in the preparation and stages of the TTM. (3) Both include components to describe or address maintenance of behaviours; through coping planning in the volition stage (HAPA) and in the maintenance stage of the TTM. 

The HAPA suggests that after individuals form intentions in the motivational stage, they enter the volitional stage to translate their set intentions into action behaviour [11]. The volitional stage requires the individual to initiate the target behaviour, to maintain ongoing behaviour, and to recover from any lapses. Past research has identified self-regulation skills (e.g., planning) as being crucial in the volitional stage to allow for the initiation and maintenance of behaviour changes in individuals [11]. 

Hence, according to the HAPA, planning assists to bridge the two stages, whereby an individuals’ intention translates to ongoing action behaviour. When engaging in planning, an individual forms an active mental representation of the target situation or behaviour they hope to achieve. Mental stimulations assist the individual to prepare for situations in which the behaviour should be performed [12], translate their formed intentions to action behaviour, and thus achieve their intended actions or goals. Past studies that examined the role of planning have often reported that planning mediated between intentions and behaviour only when individuals presented with strong behavioural intentions in the motivational stage [13]. In contrast, planning did not help to achieve action behaviour when individuals presented with weak behavioural intentions, which suggests that individuals need to set strong intentions in the motivational stage, prior to the volitional stage, to allow for actual behaviour change. 

Planning has been identified as an economical and effective way to help individuals attain their goals and to change behaviours [14]. Given the effectiveness of planning and its ease for implementation, it may be adopted as an effective way to drive physical activity-behaviour change in individuals. Planning can be categorised into two types: action planning [15] and coping planning [5,6]. 

Action planning is defined as a detailed plan that specifies one’s intentions in a ‘when’, ‘where’ and ‘how’ manner [8]. Through action planning, an individual has “a mental representation of a suitable situation (‘where’ and ‘when’) and a behavioural action (‘how’) when they form action plans” [5], which increases the effectiveness of behavioural change. We can use the example of an individual who formed the intention to run more frequently and elaborated this through an action plan to run on Mondays, Wednesdays, and Fridays at 6 pm after she ended work, for three circuits of her nearby park. Upon returning home on Monday after work, recalling her action plan would prompt her behaviour to go for a run of three circuits at her park, thus allowing her to achieve her goal of running more frequently. 

Coping planning is defined as the anticipation of difficulties or barriers that might impede one from implementing their behavioural intentions [16]. Additionally, it involves a detailed plan of how one would overcome the difficulties and barriers [5,6,17]. These detailed plans typically consist of an if-condition (prospective situation) that is linked to a then-component (behavioural response). As such, individuals anticipate potential unwanted barriers or interference (if-condition) and how they would cope with the difficulties that may interfere with the execution of the targeted behaviour (then-component) [18]. This is particularly important in familiar settings, where individuals can anticipate potential barriers. Coping planning reportedly helps to create a mental link between the foreseen barrier and a suitable alternative plan [5,6], rendering the continued performance of the behaviour more likely. When intending to perform a behaviour (e.g., running more frequently), engaging in coping planning requires the individual to develop one or more strategies (i.e., coping plans) to cope with the potentially challenging situation. Similarly, when individuals find themselves in that situation (e.g., raining after work), they would have generated potential solutions (e.g., running on the treadmill at the apartment’s gym) that would still allow them to achieve their intended behaviour. 

Although both action and coping planning are deemed as self-regulatory strategies that may facilitate behavioural action, the underlying processes of how they work also differ. Research has suggested that a combination of action and coping planning interventions for physical activity adherence has had differential effects in individuals intending to change their behaviour as compared to individuals who were already actively engaged in their intended behaviour [11,19]. A meta-analysis on the impact of action and coping planning on physical activity [20] indicates that many studies have examined the use of action planning or considered action planning and coping planning in combination. However, fewer studies have considered both action and coping planning separately within the same study. 

The effectiveness of action and coping planning in behavioural change appears to be closely dependent on whether one has set behavioural intentions and what their strength was. Despite the presence of an intention-behaviour gap, social cognitive models widely applied to health behaviours have identified that an individual’s intention remains a key determinant and strong predictor for behaviour [21]. Azjen’s theory of planned behaviour (TPB) identified that an individual’s intention to perform a behaviour (i.e., their readiness) acted as the key proximal determinant of actual behaviour [21]. 

The TPB considers ‘intentions’ under three main constructs: attitude (i.e., positive, or negative evaluation of the behaviour); subjective norm (i.e., the perception of the wishes of significant others in their lives); and perceived behavioural control (i.e., the perception of the amount of control one has for engaging in the behaviour). In a meta-analysis of 72 TPB studies on health-related exercise, Hagger et al. [22] found that intention had a medium to large correlation with exercise behaviour, where a medium to large change in an individual’s intentions to engage in exercise was associated with a small to medium sized effect change in exercise behaviour. Notably, there was significant heterogeneity in the behavioural variance accounted for by intentions across the studies analysed in the meta-analysis. This suggested that there were variations in the underlying processes that occurred after intentions were formed, where some processes allowed for physical exercise intentions to translate into actual physical exercise behaviour more strongly (i.e., no intention-behaviour gap), while other processes did not lead to actual physical exercise behaviour (i.e., having an intention-behaviour gap). 

Research on the intention-behaviour gap has suggested that the formation of intentions and the implementation of intentions into actual behaviours could be different processes [11,16]. This is consistent with the HAPA [9], which accounts for motivational and volitional stages. As such, studies have further explored variables that predicted or mediated behaviour change to equip individuals with the means necessary to bridge the intention-behaviour gap and overcome obstacles to achieve behaviour change. One key post-intentional process that explained why some individuals failed to act on their intentions was the failure to engage in planning behaviours, such as action or coping planning. Conversely, individuals who engaged in planning presented with a smaller intention-behaviour gap [13,23]. 

An individual’s motivation to engage in a behaviour could be driven by intrinsic reasons, such as gathering pleasure or satisfaction from achieving the task, or extrinsic reasons, such as the possibility of gaining a reward or consequences [24]. Past research examining the relationship between motivation and physical exercise found that higher levels of motivation were associated with greater frequency, duration, and intensity of physical exercise levels [25]. More specifically, motivation driven by intrinsic reasons promoted one’s competence, self-esteem, and wellbeing. This led to stronger intentions being formed, which thereafter translated to one initiating and maintaining the desired behaviour for a longer period. 

Habit strength has been identified as one underlying process that helps to maintain one’s behaviour [26]. Habits are developed over time when one engages in a fixed behaviour through repeated and frequent exposure to contextual or environmental cues. Over time, when a habit is being developed, an individual would automatically engage in the habitual behaviour when encountering the associated contextual and/or environmental cues [27]. For example, an individual might set intentions to improve their overall physical health, by leaving work at 6 pm and going for a run at the end of alternate workdays. Initially, the individual might require an alarm to signify the time and additional planning (e.g., to avoid scheduling dinner plans immediately after work on those days, to invite a colleague as a running companion) to help them act on their intentions and engage in the behaviour. 

The repeated behaviour of running at 6 pm on alternate workdays reinforces the mental context-behaviour association, forming a habit over time. Potentially after weeks of doing so, the individual would engage in running whenever they finish work (i.e., contextual cue), and the action eventually requires minimal forethought or planning (i.e., without the alarm to signify the time) [28]. Unlike reasoned and unfamiliar actions that require stronger effort by the individual to act on intentions, the automaticity formed habitual behaviours tends to require less cognitive demand or conscious intentions [29,30]. 

Past research on physical activity found that habits moderated the intention-behaviour relationship where the impact of intentions on behaviour diminished as habit strength increased [31,32]. Given that habit strength was more predictive of long-term action behaviour than intentions, developing a habit has been regarded as an intervention goal in behavioural change literature. Forming a strong habit also helped individuals to continue to maintain their physical activity levels even when they experienced a loss in motivation to engage in physical activity [26,33]. 

This review has identified the benefits of addressing the intention-behaviour gap and provides direction towards exploring how planning could bridge the gap and promote physical exercise in individuals. Most of the current literature that examined the use of action and coping planning to increase physical exercise targeted individuals with health conditions (e.g., cardiothoracic problems, obesity etc.) within controlled clinical settings (e.g., hospitals) through longitudinal studies [5,11,16,34]. Engaging in regular physical exercise has many short- and long-term benefits that would improve overall wellbeing and could reduce economic cost spent on healthcare. Nonetheless, there remains a high level of physical inactiveness among the general population with higher levels of inactiveness among Singaporeans as compared to global figures [35]. It remains unclear how action and coping planning may be beneficial amongst the general population who vary in their initial exercise behaviours. As compared to controlled clinical settings of hospitals, individuals within community settings with familiar daily routines and commitments may have more difficulties initiating new behaviours (i.e., increased physical exercise) and might be more aware of potential barriers when they set new intentions or plans. Potential barriers may include existing family, work or social commitments or physical tiredness after work, and thus action and coping plans would need to be set to achieve the intended behaviour. The research literature indicates that planning is an important and powerful strategy to bridge the intention-behaviour gap to allow for physical exercise behavioural change [5,6]. It is also important to note that some studies on coping planning have shown detrimental effects [36,37]. For example, individuals may become frustrated with the use of coping plans due to difficulties in foreseeing future problems and creating solutions to address them, resulting in uncompleted plans or plans of poor quality [38]. Others have indicated that action planning was ineffective at initiating behaviours under some conditions [6,11]. For example, Schroé et al. [39] found no overall effect for action planning in promoting moderate to vigorous physical activity and reducing sedentary behaviour. They suggested that individuals may not need to form plans but that the intention to engage in physical activity was sufficient to initiate past behavioural schemas. Moreover, action planning was more efficacious for individuals who were intenders (had intentions but had not executed the behaviour) versus actors (individuals who had already executed the behaviour) [39]. 

Hence, the aim of the current study was to explore whether and how action and coping planning can help to increase physical activity levels of a normal, healthy population consisting of young adults within a short time duration of two weeks. Given the suggested effectiveness of planning to support behavioural change, we hypothesised that individuals’ action and coping planning behaviours will predict an increase in physical exercise behaviours (H1). Action planning will be a stronger predictor than coping planning (H2) given the two-week time frame of the study. Consistent with past studies that highlighted the relationship between strong intentions and habits on behavioural action, we hypothesised that individual intentions to exercise (H3) and physical exercise habits (H4) will predict an increase in physical exercise behaviours.

## 2. Materials and Methods

This study was conducted as a repeated-measures design to examine how these quasi-independent variables–action planning (IV1) and coping planning (IV2)–would affect physical exercise habits in individuals. The repeated measures approach was implemented involving two time points, Time 1 (T1) at the start of the study and Time 2 (T2) at two weeks after T1. 

The primary dependent variable (DV) was change in time spent engaging in physical exercise. This was calculated by subtracting participants’ physical exercise habits (i.e., exercise behaviour in the two weeks prior to T1) from the total time spent exercising during the study’s duration of two weeks up to T2. Additionally, information about intentions to exercise (IV3), past exercise habits (IV4) and intrinsic motivation to exercise (IV5) were collected at T1 to see how these variables might be associated with physical exercise behaviours during the two weeks. 

### 2.1. Participants 

A G*Power analysis performed prior to data collection indicated that a sample of 62 participants was required, assuming a small-medium effect size at a power of 0.85. Ethics approval was gained from the university’s Ethics Committee before participant recruitment commenced (H8158). Recruitment targeted “individuals who were interested to spend more time exercising, which would benefit their health”.

A total of 92 participants were recruited at Time 1 (T1) of the study through the University’s Research Participation Management platform and through a snowballing technique via social media (e.g., Facebook). Fifteen participants indicated having previous physical injuries and all of them chose to continue with the study. Only 85 participants responded to the email sent at T2 and completed the follow-up questionnaire, resulting in an attrition rate of 7.60%. As such, data from the seven participants who did not complete the follow-up measures at T2 were excluded from the final analysis. 

The final sample consisted of 85 participants; 57.6% were female. Most participants identified as Chinese ethnicity, with a minority representation from Malay and Indian ethnicities. Of the 9.4% participants who identified as belonging to ‘other’ ethnicity, four identified as Caucasian, two as Bangladeshi, one as Burmese, and one did not specify further. Refer to Table 1 for descriptive statistics of demographic variables. Eligible student participants received partial course credit while others received neither incentive nor reward. 

### 2.2. Measures 

The Action and Coping Planning Scale (ACPS) [5,6] is a 10-item self-report measure of one’s engagement in action planning and coping planning respectively. Items were scored on a five-point Likert scale ranging from 1 (completely disagree) to 5 (completely agree). Action planning was assessed by five items: ‘*I have made a detailed plan in the next 2 weeks regarding …*’ (1) when to exercise, (2) where to exercise, (3) how I will be exercising, (4) how often I will be exercising, and (5) who I will be exercising with. Five items assessed coping planning: ‘*For the next two weeks, I have made a detailed plan regarding…*’ (1) what to do when something interferes with my plans to exercise, (2) how to cope with possible setbacks for the next two weeks, (3) what to do in difficult situations in order to act according to my intentions to increase my overall exercise time, (4) which good opportunities I could tap on to increase my exercise time, and (5) when or which situations I may have to pay extra attention to prevent lapses. During the current study, participants were also asked to state their action and coping plans at the end of each item to ensure that they had considered and thought through their responses on the Likert scale. The scores from each of the five items were summed to provide a total action planning score (IV1) and coping planning score (IV2) respectively, which reflected the participant’s engagement in each of the planning types. Sniehotta et al. [5] reported high internal consistencies with alpha coefficients between 0.92–0.95 for action planning and between 0.90–0.91 for coping planning in a sample of in-patients from rehabilitation centres. They did not report clinically meaningful cut-off scores for the measure. 

The International Physical Activity Questionnaire (IPAQ)–Short Form [40] is an international self-report measure targeting individuals aged 15 to 69. The IPAQ has good reports of test-retest reliability and high internal reliability (*α* < 0.80), with strong predictive, concurrent, convergent, criterion and discriminant validity [19,41,42], and it has been widely translated. Given this, it has been identified as suitable for use for population-based prevalence studies to examine national participation in physical activities. The IPAQ–Short Form consists of seven questions examining an individual’s seven-day recall of physical exercise across three specific intensities (i.e., walking, moderate-intensity, and vigorous-intensity) and sitting. The IPAQ defines ‘vigorous physical activity’ as activities that make you breathe much harder than normal (e.g., heavy lifting, running, fast cycling), and ‘moderate physical activity’ as activities that make you breathe somewhat harder than normal (e.g., carrying light loads, cycling). ‘Walking behaviour’ is defined as any walking to travel from place to place, or done for recreation, leisure, or exercise (minimum 10 min). These definitions were also provided to participants to help them better understand the various intensity levels. For each intensity of physical exercise, participants were required to input numerical values that indicate the frequency of their exercise behaviour (as measured by the number of days) and duration (as measured by average time in minutes spent per exercise session). 

While the order and wording of the questions remained the same for the current study, the time frame was adapted to 14 days to match the two-week study duration. Additionally, one item that asked about an individual’s time spent sitting was removed as (1) IPAQ guidelines suggest that it is not intended to be included as part of physical exercise scores, (2) its initial intended purpose was to measure sedentary behaviours, and (3) it had no specific relevance to the current study’s focus on measuring physical exercise. As advised by the IPAQ developers [43], exercise behaviour was calculated by summing the total time spent engaging in moderate and vigorous exercise behaviours (Equation (1)). While time spent walking was also recorded by participants, the data for walking were excluded from the calculations to ensure that there was a certain level of difficulty in the physical activity engaged in by participants. Time spent engaging in each exercise behaviour is calculated by multiplying the frequency (number of days) by the duration (average minutes spent on each of those days) as shown in Equation (2).

*Total physical exercise* = *Timevigorous* + *Timemoderate* (1)

*Time spent engaging in exercise* = *Frequency (days)* × *Duration (average time in minutes)* (2)

For the current study, the IPAQ–Short form was used to measure participants’ total (1) intentions to exercise in the two-week period of the study (IV3), (2) past exercise habits in the two weeks prior to the start of the study (IV4), and (3) actual time spent engaging in vigorous and moderate physical exercise during the two weeks of the study. The dependent variable, change in exercise time (between T2 and T1), was measured by subtracting (2) from (3). 

Intentions to exercise (IV3) refers to the total physical exercise time an individual intends to engage in vigorous and moderate physical exercise in the upcoming two weeks as per the study’s duration. This allowed us to understand whether participants’ initial intentions predicted their increase in physical exercise time at T2, as highlighted in past research [44]. The IPAQ–Short Form was adapted to allow us to measure an individual’s intentions to exercise. Instead of asking participants to recall how much they had exercised in the past two weeks, participants were asked about the frequency and duration with respect to their stated intentions to exercise for the following two weeks *(‘During the next 14 days, how many days do you intend to do vigorous physical activities?’*). This method to assess an individual’s intention to engage in physical exercise was similarly utilised in past literature [44,45]. 

The Intrinsic Motivation Inventory (IMI) measures one’s subjective experience towards a target activity [46,47]. Each item is rated on a seven-point Likert scale from 1 (not at all true) to 7 (very true). Although the IMI consists of a total of 45-items in six subscales, the original scale developers acknowledged that items within the subscales overlap considerably and subscales appear to be independent of others and thus could be eliminated based on sample needs and clinical utility [47,48]. Other studies have also made adaptations to the inventory to better fit their studies [49]. As such, the current study focused on ten items based on their specific relevance–four (of 7) from the subscale of value/usefulness and six (of 7) from interest/enjoyment. The subscales for perceived competence, effort/importance, pressure/tension, perceived choice, and relatedness were excluded as they were not relevant to the current study. McAuley et al. [48] reported that all subscales had acceptable Cronbach’s alpha internal consistencies between 0.68 to 0.87. Of the ten items, one item was reversed scored given that it was worded in a “negative” way. The ten item scores are summed to provide a total score for ‘motivation to exercise’. A higher total score would reflect greater intrinsic motivation to engage in exercise. 

Demographic information of participants such as age, gender, race and previous health conditions or injuries that may affect exercise were also captured at the start of the study during T1. 

Concluding the study at T2, participants were asked to rate their perceived effectiveness of the planning components in the current study (I feel that my planning at the start of the 2 weeks has helped me to increase the amount of time I engage in physical exercise), their confidence to maintain their physical exercise levels reflected during the study (I am confident that I will be able to continue exercising at the rate I have done for the past two weeks), and to report if they were in the habit of planning for their exercises prior to the study (I am usually in the habit of making plans to exercise even before participating in this study). Participants responded using a seven-point Likert scale ranging from 1 (not true at all) to 7 (very true). These questions were administered to better understand whether participants felt that planning was beneficial as well as the sustainability of their exercise behaviours beyond the study. Additionally, having participants report if they were in the habit of planning prior to the study may help to explain potential results of the study. 

### 2.3. Procedure 

The study was delivered through Qualtrics software, Version 2020 (Qualtrics, Provo, UT, https://www.qualtrics.com) (Accessed on 13 November 2020). To reduce attrition rates, participants were sent a reminder email through Qualtrics at the mid-point of the study (i.e., one week after T1) to encourage them to continue their exercise plans according to their intentions and plans stated at T1. Participants were provided with summary information about the study in a prefacing page to the questionnaires. Participants were invited to provide informed consent to participate, verify they were above 18 years old, confirm that they were keen to increase their physical activity levels, and agree to complete the measures at both T1 and T2 as well as to be contacted with reminder emails containing instructions. 

The T1 measures were the IPAQ past 14 days, IPAQ intentions next 14 days, ACPS, elaborate their plans, IMI, and demographics including past and/or current injuries. The T2 measures were the IPAQ past 14 days, perceived effectiveness of planning, and confidence in continuing their levels of physical exercise beyond T2. They were also given an option to opt out of the study without penalty and asked if they had answered truthfully at the end of the questionnaire for both time points. 

### 2.4. Data Cleaning and Analysis 

Data cleaning and analyses were carried out using SPSS Version 26 software. Cronbach’s alpha tests were carried out to examine reliability for each of the scale measures. A hierarchal multiple regression was thereafter conducted to investigate the contribution of motivation to exercise (step 1), intentions (step 2), past exercise habits (step 3), action planning (step 4), and coping planning (step 5) in predicting change in exercise time. A further *t*-test analysis examined the differences in planning behaviours between participants who increased in their exercise behaviours to meet recommended guidelines for physical exercise and those who continued to fail to do so. Alpha was set to 0.05 for all analyses. 

## 3. Results

At T1, 58.8% of participants reported physical exercise levels that met recommended guidelines (i.e., completing ≥150 min of moderate intensity or ≥75 min of vigorous intensity, or an equivalent combination of moderate and vigorous intensity) in the two weeks prior to the time of reporting. At T2, there was a 13.0% increase where 71.8% of participants reported physical exercise levels that met recommended guidelines across the two-week period of the study. Of the 15 participants who indicated having previous physical injuries, descriptive statistics showed an overall positive change in exercise behaviour across the two time points (*M* = 27.7, *SD* = 427.1), with 80.0% meeting recommended guidelines at T2. This pattern of results appears consistent with other participants, suggesting that their past injuries did not negatively bias the study’s results. 

### 3.1. Reliability Analyses 

Cronbach’s alpha analyses were carried out to calculate the respective internal consistency of the Intrinsic Motivation Inventory (IMI) (∝ = 0.86), the adapted International Physical Activity Questionnaire (IPAQ) (α = 0.74), the Action planning scale (∝ = 0.89), and Coping planning scale (∝ = 0.85). There was a high level of internal consistency for the respective measures which suggests that all items included in each measure reliably predicted for the variable examined. For the IPAQ, we also calculated nonparametric Spearman correlation coefficients of test-retest agreement for time 1 and time 2 measurements [41]. Spearman’s ρ coefficients were all significant with repeatability values as follows for vigorous physical exercise days (0.766) and time (0.688), moderate exercise days (0.557) and time (0.241) and walking days (0.672) and time (0.403). Table 2 includes the descriptive statistics for all measures. 

### 3.2. Primary Analysis of Hierarchal Multiple Regression 

#### 3.2.1. Assumptions Testing 

Prior to running the primary analysis, we evaluated the assumptions for a hierarchal multiple regression. There was independence of residuals, as assessed by a Durbin–Watson statistic of 1.646. Scatterplots indicated a linear relationship between the dependent variable and the four independent variables individually and collectively. A visual inspection of the plot of standardized residuals versus unstandardized predicted values revealed homoscedasticity. No multicollinearity was observed as all tolerance and VIF scores were greater than 0.1 and less than 10, respectively. Casewise diagnostics identified one case that may act as an outlier and two other cases with potentially risky leverage scores (i.e., leverage scores between 0.2 and 0.5). However, these cases were not identified as significant influential points (all Cook’s distance values < 1.0). Additionally, closer examination of the data revealed that these cases reported engaging consistently in physical exercise across the two weeks. Hence, the extreme predictor values (i.e., exercise time at T2) were likely because they engaged in physical exercise more or less frequently than initially intended, instead of them engaging in a once-off unusual exercise behaviour (e.g., hiking) which would confound the pattern of results. Hence these cases were not removed from the overall analysis. No cases were identified to be influential points as Cook’s distance values were all below 1. Normal Q-Q plot of the standardized residuals showed that residual errors were approximately normally distributed. To summarise, assumptions were met, and all 85 participant cases were included in the regression model. 

#### 3.2.2. Regression Model

A hierarchical multiple regression was conducted to examine how motivation, intentions to exercise, prior exercise habits, action planning, and coping planning predicted for change in exercise behaviour at T2. Refer to Table 3 for the hierarchal regression model results. The full model was statistically significant, *F*(5, 79) = 12.93, *p* < 0.001; adjusted *R*^2^ = 0.42. The full model (Model 5) accounted for 45.0% of variability. Motivation was entered into the model to control for participants’ motivations to increase their time spent exercising. The regression analysis indicated that motivation did not significantly predict for change at T2 (Model 1). Participants’ motivation levels were all above the mid-range score indicating consistently moderate to high motivation to increase exercise behaviour across the sample. 

Intentions significantly predicted for change in exercise behaviour at T2 (Model 2). In Model 3, the addition of prior physical exercise habits led to a statistically significant increase in *R*^2^ of 0.14, (3, 81) = 18.87, *p* < 0.001. However, the addition of physical exercise habits in the model resulted in intentions no longer being a significant predictor. In Model 4, action planning was a significant predictor for change in exercise behaviour even after accounting for past exercise habits, *F*(4, 80) = 15.96, *p* < 0.05, Δ*R*^2^ = 0.05. However, the addition of coping planning to predict for change in exercise behaviour (Model 5) was not significant, *F*(5, 79) = 12.93, *p* > 0.05, Δ*R*^2^ = 0.01. 

#### 3.2.3. *t*-Test Analysis for Action and Coping Planning Scores

Further analysis was conducted for a subgroup of participants. To understand whether participants had met WHO guidelines, their individual PA scores were calculated and compared to the WHO PA guideline of 150 min of physical exercise per week before being assigned to either Met or Not Met groups. Vigorous exercise scores were multiplied by 2 to match the intensity of moderate exercises and added together to their respective time spent on moderate exercise and the final values were halved to match a 1 week duration: ((VigourousEx × 2) + (ModerateEx)/2). A Chi-Square test showed a significant association between time of measurement and proportion of participants meeting the WHO recommended guidelines (Χ2(1) = 24.54, *p* < 0.001); 71.8% of participants met the WHO recommended guidelines for physical activity at the end of the study compared to the initial 58.8%, which evidences an increase in participants’ physical exercise behaviours. 

Participants who failed to meet WHO recommended guidelines for physical exercise at T1 (*n* = 35) were further grouped into two groups: those who subsequently met recommended guidelines at T2, and those who continued to fail to meet recommended guidelines at T2. Prior to conducting the analysis, necessary assumptions for *t*-test analysis were tested. The boxplot indicated no extreme outliers in the data, and data were normally distributed as assessed by Shapiro–Wilk’s test (*p* > 0.05). There was homogeneity of variance as assessed by Levene’s test for equality of variances (*p* = 0.875 and 0.309 respectively for action and coping planning).

**Figure 1:**
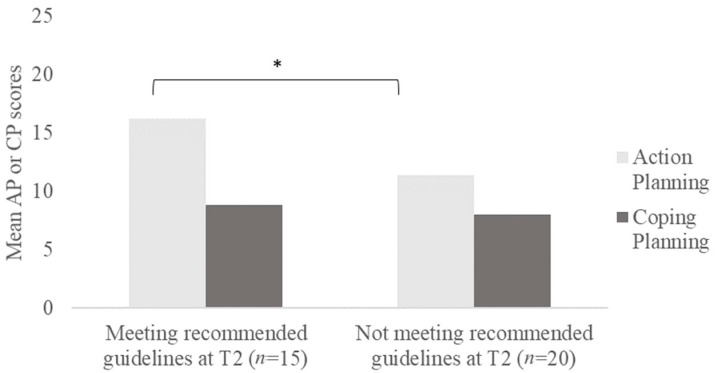
Differences in mean action and coping planning scores for participants who did not meet recommended physical exercise guidelines at time point 1 (*n* = 35). Note. * = statistically significant difference at *p* < 0.05 level.

A *t*-test was conducted to identify if there were any significant differences in action planning and coping planning scores between these two sample groups (see Figure 1). There was a statistically significant difference in action planning scores between the two groups, *t* (33) = 2.631, <0.05, 95% CI (1.10, 8.60). Individuals who subsequently met recommended guidelines at T2 had significantly higher action planning scores (*M* = 16.2, *SD* = 5.0) compared to individuals who continued to not meet recommended guidelines for physical exercise (*M* = 11.4, *SD* = 5.6). In contrast, there was no significant difference in coping planning scores between the two groups, *t* (33) = 0.60, >0.05, 95% CI (−1.96, 3.59). Coping planning scores were similar between those who increased their physical exercise behaviour at T2 (*M* = 8.9, *SD* = 3.5) and those who maintained low levels of physical exercise behaviours (*M* = 8.1, *SD* = 4.3).

### 3.3. Supplementary Questions Examining Participants’ Perspectives

Lastly, we examined participants’ perspectives on whether they found the planning components in the study effective, their confidence to maintain their physical exercise levels reflected during the study, and whether they were in the habit of planning for their exercise prior to the study. On average, we found that participants reported confidence in maintaining physical exercise and had some prior habits of planning prior to this study. However, despite our findings that suggested the effectiveness of planning in predicting for change in exercise behaviours, participants rated the planning components as effective to only a moderate degree.

As seen from Table 2, the maximum amount of time recorded for participants’ past habits of time engaging in moderate and physical exercise was a total of 2520 min across two weeks. The time here equates to an average of three hours of physical exercise of moderate- or vigorous-intensity daily which seems unlikely for most individuals unless they were training as an athlete. Open-ended responses revealed that one participant indicated carrying light loads and brisk walking for three-hours daily. It is hypothesised that this pattern of exercise behaviour may be related to the participant’s daily responsibilities (e.g., delivery services, jobs that require manual labour, or caring for a young child). Hence large variability in results obtained may also be attributed to participants’ daily responsibilities and jobs that are physically demanding, rather than intentional physical exercise behaviours.

## 4. Discussion

The current study aimed to explore whether and how action and coping planning can help to increase physical activity levels of a normal healthy population consisting of young adults within a short time duration of two weeks. We explore our findings in relation to the four hypotheses.

### 4.1. Summary of Key Findings

Firstly, this study examined how an individual’s action planning and coping planning behaviours affected physical exercise behaviours. It was hypothesised that both action planning and coping planning would predict for an increase in time spent engaging in exercise behaviours, and more specifically action planning would be a stronger predictor than coping planning. The former hypothesis was only partially supported. Action planning significantly explained for an increased variance when predicting for change in exercise behaviour. However, coping planning did not further strengthen the model predicting for change. This finding was supportive of our latter hypothesis, which suggested that action planning was more effective than coping planning.

Secondly, we hypothesised that an individual’s intentions to increase their time engaged in physical exercise would result in an actual increase in physical exercise behaviours in the two-week study. This hypothesis was supported. Intentions to exercise accounted for the largest variance when predicting for the change in exercise behaviour over the two-week study. This suggested that individuals who had strong intentions to increase their physical exercise were more likely to initiate an increase of and/or maintain their physical exercise behaviour. This finding is consistent with past studies that showed that intentions could translate to actual physical exercise behaviour [21,22]. However, it appears that the relationship between intentions and the change in exercise behaviour is effective only in the absence of past exercise habits.

Thirdly, we hypothesised that physical exercise habits will predict for an increase in physical exercise behaviours in the two-week study. As hypothesized, an individuals’ past habits for physical exercise significantly predicted for an increase in exercise time at T2. This suggests that individuals who had strong habits for physical exercise prior to the study spent more time engaging in physical exercise in the two-week study period. Individuals’ past habits accounted for the second largest variance (after intentions to exercise) when predicting for an increase in time spent engaging in physical exercise. The findings here were consistent with previous literature and our hypothesis that an individual’s habits would be able to predict for their physical exercise behaviours.

### 4.2. Overall Increase in Exercise Behaviours in Our Study

Our results indicated that our two-week study supported individuals in increasing their exercise behaviours. The end of the two-week study saw that 71.8% of our participants now met the WHO recommended physical activity guidelines. This figure is a 13.0% increase from our findings at time-point 1 and, of note, higher than past findings where only 63.5% of Singaporean adults were physically active [1]. This figure is also closer to global figures where three in four (i.e., 72.5%) would be physically active. While of a small value, we also saw a mean increase in exercise time of 15.3 min over the two-week study.

#### 4.2.1. Action Planning to Initiate Behaviour Change

Action planning was found to be predictive of an increase in physical exercise behaviours across our sample. This suggested that the effectiveness of action planning was not only limited to participants who were physically inactive. This contrasted with the findings reported by Scholz et al. [11], who suggested that action planning was not beneficial for participants who were already physically active. Utilising action plans, individuals form a mental representation of the ‘where’, ‘when’ and ‘how’ of achieving their intended behaviours. This likely increased the saliency of the steps needed by individuals to increase their overall exercise behaviours [8]. Consistent with past studies [13,50] and the HAPA model [9], our findings affirmed the effectiveness of action planning to bridge the intention-behaviour gap.

Action planning was particularly beneficial for inactive participants with high intentions to increase physical exercise behaviours and less so for inactive participants with low intentions. This emphasises the role of action planning in behavioural initiation and aligns with literature reporting that the effects of intentions to predict for behaviour tend to diminish when habit strength increases [31,32]. Hence, developing habits to exercise will more likely lead to the promotion and sustenance of exercise behaviours. Action planning was adopted in the post-intentional stage, allowing for behavioural intentions to translate into actual physical exercise behaviour, bridging the intention-behaviour gap [16,51,52]. Engaging in action planning also allowed for a 13.0% increase in the number of participants to meet WHO recommended guidelines for physical activity.

Given the effectiveness of action planning observed in our study regardless of participants’ initial physical exercise levels, it may be used as an effective intervention tool at an individual level. This would help individuals with intentions to be more physically active, translate their intentions into actual behaviours and, over time, possibly develop strong habits for exercise.

#### 4.2.2. Coping Planning Unable to Help Individuals Increase Exercise Behaviours

Coping planning did not help participants increase their time spent engaging in physical exercise. Making coping plans requires individuals to anticipate barriers and difficulties that may hinder them from engaging in physical exercise behaviours and has more commonly been found to be helpful for behavioural maintenance [5,6,17]. There are a few potential explanations as to why coping planning was not found to be predictive.

Firstly, our study’s aim was for participants to increase their time spent engaging in physical exercise. This assumes that participants are engaging in behavioural initiation instead of behavioural maintenance, and hence action planning may be more effective. Further, given the large variability in pre-existing exercise behaviours, with some participants reporting three hours a day on exercise behaviours, it was likely that a further increase in exercise behaviours was unlikely for some.

Secondly, it was noted that the mean score for coping planning was much lower than for action planning, with participants reporting that they generally do not engage in coping plans when setting behavioural intentions and plans. This may be related to a preference to not consider possible barriers and obstacles as an increased saliency of these thoughts may reduce one’s motivation and self-efficacy to engage in physical exercise [36,37]. For example, if an individual anticipates that they may not be able to go to the gym because they may be physically tired after the workday, the thought of the barrier may decrease their motivation and perceived ability to engage and accomplish their task [36]. This leads to a failure to engage in their intended behaviours.

Thirdly, it may be that participants had already anticipated potential barriers when making their action plans. Given that our study used a community sample, participants should already be familiar with their daily routines and commitments and may not have to intentionally consider potential obstacles or make if-then plans [18]. For example, an individual who is already familiar with their family’s habits of gathering for dinner on one of the weekend days may intentionally set action plans to exercise on weekdays rather than weekends. This would not require them to consider coping plans if a family dinner appointment unexpectedly interferes with their plans to exercise.

Finally, it may be that coping planning would be more effective over a longer duration. Sniehotta et al. [5] assumed that action planning is the more important strategy in earlier stages of behaviour change, whereas coping planning might be more important in later stages. A longer intervention period would be required to test this. In summary, our study did not find an effect of coping planning on a prediction for an increase in exercise behaviours. However, our pattern of results is consistent with past studies which suggested that (a) planning was an effective way to help individuals attain behavioural change, and (b) the processes underlying action and coping planning differed.

#### 4.2.3. Comparison with Singaporeans’ Exercise Behaviours

A total of 58.8% of the participants who participated in the study were already in the habit of engaging in regular physical exercise in accordance with the WHO recommended guidelines for physical activity [35]. This meant that per week, these participants were already meeting 150 min of moderate- to vigorous-intensity physical activity, 75 min of vigorous-intensity physical exercise or a combination of both. The remaining 42.1% of participants who did not meet WHO guidelines were higher than the 36.5% reported by a study conducted by the WHO for Singaporeans [1]. This suggested that our current sample tended be less physically active than the overall Singapore population.

It is relatively unexpected for our study to have a lower percentage of individuals meeting recommended guidelines compared to the population statistics. Participants’ mean age suggested that many were young adults who were either studying or who were in their early careers. The literature has identified that people in the age group between 18 to 44 years were more likely to exercise than other older age groups [53]. It is possible that the discrepant findings were due to common barriers such as poor time management, lack of access to facilities, or increased commitments or priorities [7,54] that were further exacerbated due to the COVID-19 pandemic.

Due to circuit breaker and safe-distancing measures employed for COVID management, participants may have faced disrupted routines, increased sedentary lifestyles, and unexpected barriers to engage in physical exercise [35,55,56]. For example, individuals may have had to work within a limited space or with exercise equipment available at home due to the closure of public and private fitness facilities. They could also have faced increased familial commitments with family members spending more time at home or struggled with their mental health and consequent impairments on daily functioning. Additionally, during the data collection period, Sport Singapore announced stringent measures while exercising, which included having to maintain a safe distance and wearing a mask during warm-up or even during exercise, with the exception of strenuous exercises. Failure to do so may have incurred hefty fines and even a prosecution in court [57]. These were unanticipated barriers that could have resulted in less consistent or less frequent exercise behaviours during the period of our study, as usual habits were disrupted, and individuals had to adapt to new routines.

### 4.3. Limitations and Future Directions

It is also important to consider our study’s limitations. Firstly, this study was carried out during the COVID-19 pandemic. We must acknowledge that the significant disruption in daily routines due to the measures put in place may have potentially confounded the study’s results. Participants may have faced new motivators or barriers while adapting to new routines (e.g., disruption in exercise behaviours, increased opportunities due to online fitness workouts). These may bridge or further widen the intention-behaviour gap.

Secondly, the data and standard deviations reflect a large variability in participants’ past exercise habits at T1 which could lead to a couple of confounding factors that may impact the accuracy of the study. Participants with strong exercise habits or who were already meeting WHO recommended guidelines at T1 may have presented with a ceiling effect; it may not have been realistic for these participants to further increase the amount of time they could possibly engage in physical exercise during the study. While focusing still on a community sample, future studies could recruit participants with low exercise habits or examine participants who were interested in engaging in new exercise behaviours (e.g., learning a new sport). Additionally, it could be helpful to include a question to examine participants’ perspectives on how much they felt they were exercising as compared to others.

Thirdly, it will be helpful to have a consistent definition of what constitutes exercise behaviours. Despite the use of the IPAQ measure that defined exercise behaviours, the IPAQ did not exclude one-off exercise behaviours (e.g., hikes) that may not be a routine behaviour and it is unable to differentiate between leisure physical exercise as compared to daily activities and/or employment that may be physically demanding. Hence, this limits the generalisability of our current findings. Additionally, given the habitual nature of daily activities, action and coping planning might not improve behavioural initiation or maintenance.

Fourthly, our study was of a relatively short timeframe of two weeks, which may not be sufficient to examine behaviour change over time. Additionally, it is important for individuals to sustain their engagement in regular physical exercise to reap its long-term benefits. Future studies could consider employing a longer duration of a few months to better capture consistent physical exercise behaviours, examine the effects of coping planning, and to examine whether behavioural change is sustained.

Lastly, given the positive results of the current study, it would be helpful if future studies adopted a between-within groups design to examine action planning and coping planning as an intervention. Experimental procedure could include the experimenter prompting participants to consider and make action and/or coping plans. This design was not possible in the current study given the restrictions of face-to-face contacts given the COVID-19 situation. However, this would allow for better understanding of the differing functions of action and coping planning and provide evidence for whether coping planning may have detrimental effects on specific groups of participants.

## 5. Conclusions

Given the benefits of physical exercise on both physiological and psychological domains, Singapore has committed to increasing overall physical activity levels across all age groups. However, behavioural change has its own challenges. This study explored how action and coping planning could help bridge the intention-behaviour gap and increase physical exercise behaviours. The present study identified action planning as an effective post-intentional self-regulatory strategy to allow for behavioural change. While action planning was helpful, it is also worth noting that an individual’s intentions and past habits for physical exercise remained as strong predictors for any increase in exercise behaviour. Together, the findings suggest that government-wide approaches to target physical inactivity within the population should also differentially target population groups with different presenting behaviours. Planning appears to be more helpful at the individual micro-level. Specifically, action planning could target participants who were not meeting recommended guidelines or those who would like to engage in new physical exercise behaviours and who have set strong intentions in the motivational stages. Given that it is a cheap yet effective strategy to bridge the intention-behaviour gap, action planning could be widely implemented. This could help more individuals increase their physical exercise behaviours, develop good habits, and reap longer-term health benefits. Nationwide, this would lead to a more productive and healthier population, with lower economic costs on public health.

## Figures and Tables

**Table 1:** Descriptive statistics of demographic variables.

Variable	Female (*n* = 49)		Male (*n* = 36)		Overall Sample (*n* = 85)	
Age						
Mean (sd)	25.7 (7.07)		26.1 (5.86)		25.8 (6.54)	
Range	18–55		19–56		18–56	
	*n*	%	*n*	%	*n*	%
Ethnic representation						
Chinese	40	81.6	32	88.9	72	84.7
Malay	2	4.1	1	2.8	3	3.5
Indian	2	4.1	-	-	2	2.4
Other	5	10.2	3	8.3	8	9.4
Presence of past injuries	6	12.2	9	25.0	15	17.6
Met WHO recommend						
Time point 1	29	59.2	21	58.3	50	58.8
Time point 2	35	71.4	26	72.8	61	71.8

**Table 2:** Descriptive statistics for measures.

	Possible Response Range	M	SD	Min	Max
Motivation to exercise	10.0–70.0	59.9	6.9	41.0	70.0
Action planning scores	5.0–25.0	16.6	5.8	5.0	25.0
Coping planning scores	5.0–25.0	10.2	5.0	5.0	25.0
Past exercise habits (T1–14 days)					
Moderate exercise					
Frequency (days)	0.0–14.0	2.6	3.0	0.0	14.0
Duration per session (min)	≥0	28.0	34.4	0.0	180.0
Total time (min)		113.8	290.7	0.0	2520.0
Vigorous exercise					
Frequency (days)	0.0–14.0	4.3	3.6	0.0	14.0
Duration per session (min)	≥0	44.7	35.3	0.0	150.0
Total time (min)		250.6	278.4	0.0	1260.0
Walking		310.8	443.9	0.0	1980.0
Moderate + Vigorous (min)		364.4	385.1	0.0	2520.0
Intentions to exercise					
Moderate exercise					
Frequency (days)	0.0–14.0	3.9	3.7	0.0	14.0
Duration per session (min)	≥0	32.6	33.6	0.0	180.0
Total time (min)		159.8	290.3	0.0	2520.0
Vigorous exercise					
Frequency (days)	0.0–14.0	5.6	3.6	0.0	14.0
Duration per session (min)	≥0	24.9	31.6	0.0	150.0
Total time (min)		288.0	279.1	0.0	1440.0
Walking		288.0	343.0	0.0	1680.0
Moderate + Vigorous (min)		447.8	387.3	0.0	2520.0
Actual exercise behaviour (T2)					
Moderate exercise					
Frequency (days)	0.0–14.0	3.5	3.3	0.0	14.0
Duration per session (min)	≥0	39.1	42.7	0.0	200.0
Total time (min)		153.2	203.8	0.0	1400.0
Vigorous exercise					
Frequency (days)	0.0–14.0	4.3	3.4	0.0	12.0
Duration per session (min)	≥0	41.3	33.8	0.0	180.0
Total time (min)		226.6	235.0	0.0	1080.0
Walking		313.0	394.0	0.0	2100.0
Moderate + Vigorous (min)		379.7	306.6	0.0	1400.0
Change in exercise behaviour (T2-T1)					
Moderate exercise (min)		39.4	209.4	−1120.0	690.0
Vigorous exercise (min)		−24.1	173.1	−720.0	360.0
Moderate + Vigorous (min)		15.3	274.9	−1120.0	930.0
Effectiveness of planning	1.0–7.0	3.8	1.7	1.0	7.0
Confidence to continue exercise behaviour	1.0–7.0	4.7	1.6	1.0	7.0
Prior habit of planning	1.0–7.0	4.3	1.9	1.0	7.0

Note. This table shows the specific descriptive statistics for each variable measured in the current study. Total time (min) refers to the mean total time spent exercising by each participant, as calculated by multiplying each participant’s frequency of exercise by the duration per session (see methods session for the formula).

**Table 3:** Hierarchal multiple regression model for change in exercise time (T2-T1).

Model	Variable	$R^{2}$	$\mathrm{Adj}\;R^{2}$	$\Delta R^{2}$	B	SE B	β	*t*	*p*	95% CI
1	Motivation	0.01	−0.01	0.01	3.20	4.34	0.08	0.74	0.46	−0.54, 11.8
2	Motivation	0.27	0.26	0.27 ^	5.48	3.76	0.14	1.46	0.15	−1.99, 12.9
	Intentions				−0.37	0.07	−0.52	−5.47	0.00 **	−0.50, −0.23
3	Motivation	0.41	0.39	0.14 ^	5.16	3.40	0.13	1.52	0.13	−1.61, 11.9
	Intentions				0.22	0.15	0.31	1.49	0.14	−0.08, 0.51
	Habits				−0.65	0.15	−0.91	−4.38	0.00 **	−0.94, −0.35
4	Motivation	0.44	0.42	0.03 ^	2.23	3.59	0.06	0.62	0.54	−4.91, 9.38
	Intentions				0.22	0.14	0.30	1.54	0.14	−0.07, 0.50
	Habits				−0.70	0.15	−0.98	−4.84	0.00 **	−0.99, −0.41
	Action Planning				9.98	4.62	0.21	2.60	0.03 *	0.79, 19.2
5	Motivation	0.45	0.42	0.01	2.95	3.68	0.07	0.80	0.42	−4.36, 10.3
	Intentions				0.24	0.15	0.34	1.65	0.10	−0.05, 0.54
	Habits				−0.72	0.15	−1.01	−4.84	0.00 **	−1.01, −0.42
	Action Planning				1254	5.35	0.26	2.34	0.02 *	1.85, 23.2
	Coping Planning				−5.69	6.09	−0.10	−0.94	0.35	−17.8, 6.42

Note. * = significant at *p* < 0.05 level, ** = statistically significant at *p* < 0.001 level, ^ = significant $\Delta R^{2}$.

## Data Availability

Data are available upon request from the corresponding author.
